# Endectocides as a complementary intervention in the malaria control program: a systematic review

**DOI:** 10.1186/s13643-021-01578-9

**Published:** 2021-01-18

**Authors:** Fereshteh Ghahvechi Khaligh, Abbas Jafari, Elena Silivanova, Mikhail Levchenko, Bahlol Rahimi, Saber Gholizadeh

**Affiliations:** 1 Social Determinants of Health Research Center, Clinical Research Institute, Urmia University of Medical Sciences, Urmia, Iran; 2 Medical Entomology Department, School of Public Health, Urmia University of Medical Sciences, Urmia, Iran; 3 Department of Clinical Toxicology, School of Medicine, Urmia University of Medical Sciences, Urmia, Iran; 4 Cellular and Molecular Research Center, Research Institute on Cellular and Molecular Medicine, Urmia University of Medical Sciences, Urmia, Iran; 5 All-Russian Scientific Research Institute of Veterinary Entomology and Arachnology, Branch of Federal State Institution Federal Research Centre Tyumen Scientific Centre, Siberian Branch of the Russian Academy of Sciences (ASRIVEA – Branch of Tyumen Scientific Centre SB RAS), Institutskaya st. 2, Tyumen, Russian Federation 625041; 6 Department of Health Information Technology, School of Applied Medical Sciences, Urmia University of Medical Sciences, Urmia, Iran

**Keywords:** Ivermectin, Endectocides, Systemic insecticides, Malaria elimination

## Abstract

**Background:**

Malaria is the most common vector-borne disease transmitted to humans by *Anopheles* mosquitoes. Endectocides and especially ivermectin will be available as a vector control tool soon. The current review could be valuable for trial design and clinical studies to control malaria transmission.

**Methods:**

PubMed/MEDLINE, Scopus, Web of Science, and Science Direct were searched for original English published papers on (“Malaria chemical control” OR “Malaria elimination” OR “*Anopheles* vector control” OR “Malaria zooprophylaxis”) AND (“Systemic insecticides” OR “Endectocides” OR “Ivermectin”). The last search was from 19 June 2019 to 31 December 2019. It was updated on 17 November 2020. Two reviewers (SG and FGK) independently reviewed abstracts and full-text articles. Data were extracted by one person and checked by another. As meta-analyses were not possible, a qualitative summary of results was performed.

**Results:**

Thirty-six published papers have used systemic insecticides/endectocides for mosquito control. Most of the studies (56.75%) were done on *Anopheles gambiae* complex species on doses from 150 μg/kg to 400 μg/kg in several studies. Target hosts for employing systemic insecticides/drugs were animals (44.2%, including rabbit, cattle, pig, and livestock) and humans (32.35%).

**Conclusions:**

Laboratory and field studies have highlighted the potential of endectocides in malaria control. Ivermectin and other endectocides could soon serve as novel malaria transmission control tools by reducing the longevity of *Anopheles* mosquitoes that feed on treated hosts, potentially decreasing *Plasmodium* parasite transmission when used as mass drug administration (MDA).

**Supplementary Information:**

The online version contains supplementary material available at 10.1186/s13643-021-01578-9.

## Background

Malaria is a parasitic infectious disease of poverty and one of the major global public health problems [1]. Long-lasting insecticidal nets (LLINs), IRS, artemisinin combination therapy, transmission-blocking vaccines, the deployment of single, low-dose primaquine, and antimalarial drugs are several WHO-recommended strategies in reducing the burden of malaria [2, 3]. The widespread and sustained use of pesticides for malaria control has resulted in varied environmental and entomological issues mainly the selection of *Anopheles* mosquitoes for resistance to the primary vector control strategies [4, 5]. Systemic insecticides are favorable solutions against insecticide-resistant and zoophilic/zoophagic mosquitoes [6].

Systemic insecticides are applied in veterinary, horticulture [7], and recent medical entomology [8]. Reports on the development of resistance to systemic insecticides in pests are scarce [9, 10]. Among the 21 vector control tools evaluated in a review (shrinkingthemalariamap.org), less than 10 were supported by phase II or phase I evaluation of ivermectin, fipronil, and eprinomectin as endectocide administration in humans/animals, showing the rich pipeline of research into them at earlier stages of evaluation [11]. Endectocides are drugs applied directly to hosts to kill endoparasites and ectoparasites, mainly blood-feeding arthropods [12]. Although being used for the control of nematodes in humans and other vertebrates, endectocides can be toxic to *Anopheles* spp. when mosquitoes feed on a host recently received these drugs [13].

There are various advantages for systemic insecticides, including commercially available and easily accessible, relatively inexpensive, easy to administer in oral formulations, long-lasting, circulate uniformly in the blood for consistent vector uptake, have good safety, collateral benefits to treated animals [14]. However, the main concern in the wider scale application of them is related to the risk assessment of systemic insecticides in ecosystem functioning and services. There are several examples of the negative impact of these compounds on decomposition, nutrient cycling, soil respiration, and invertebrate population [15].

The role of livestock in malaria epidemiology is diverting malaria vectors to dead-end host and prevent parasite amplification by zooprophylaxis [16]. For this purpose, animals must be kept close to the man; therefore, it can increase malaria transmission by zoopotentiation, increasing the numbers of mosquitoes by keeping animals close to humans [16, 17] in areas where cattle production is semi-intensive or semi-extensive. However, social local factors in animal husbandry, scale of animal husbandry, and subsequent influence on the potential use of the endectocides need to be considered. The use of endectocides treated cattle as complementary intervention highly attractive in areas where humans and animals are close together, especially when the malaria mosquito is zoophagic such as *An. arabiensis* [18].

In the current review, published papers related to the application of systemic insecticides and endectocides for malaria vectors and parasites and their potential role as a new intervention for malaria elimination are discussed.

## Methods

The protocol of the current study was conducted according to the checklist and guidelines of the Preferred Reporting Items for Systematic Reviews and Meta-Analyses (PRISMA) statement [19] (Supp. 1). Electronic databases, such as MEDLINE via PubMed, Web of Science, and Scopus, were searched for relevant primary studies until 2019. English language publications were eligible and conference abstracts were not eligible for inclusion. The selection of studies based on their title and abstract was performed using Endnote X9 (Bld12062) and full-text publication was reviewed carefully. Eligibility assessment was performed independently in an unblinded standardized manner by two reviewers. We developed a data extraction sheet (Table 2), one review author (FG) extracted the following data from the included studies, and the second author (SG) checked the extracted data. Duplicate and ineligible studies were eliminated from further review. Disagreement on the eligibility of studies was resolved by discussion or consensus. A limited update literature search was performed on 17 November 2020. Although we had planned to assess reporting bias, there were too few included studies considering the same intervention to allow this to be done.

Information was extracted from each paper on (1) *Anopheles* species and the inclusion and exclusion criteria; (2) type of intervention including the type of systemic insecticides, dose, and host; and (3) type of outcome measure including survival rate, sporontocidal effect, reproduction rate, and malaria transmission.

The relative risk of mosquito mortality increasing, egg production reduction, and malaria transmission reduction was the primary measure of treatment effect. The nature of the included studies meant that much of the analysis was anticipated to be narrative. The data of studies were not combined and assume no serious inconsistency.

## Results

The searches included 117 published papers on systemic insecticides and malaria control. Eight insecticides were applied mostly in different studies (Table 1). Doramectin, moxidectin, eprinomectin, and ivermectin (IVM) were used as primary endectocides in malaria control studies. IVM was also assessed as a systemic insecticide in 74% of the extracted papers on vector control.

**Table 1:** Details of components used as systemic insecticides in this review

Insecticides	Chemical class	Mode of action	Reference
Afoxolaner	Isoxazolines	Antagonists of GABA- and glutamate-gated chloride channels.	[14]
Fluralaner			[20]
Sarolaner			[21, 22]
Diflubenzuron	IGR	Involves inhibiting the production of chitin	[12]
Eprinomectin	Macrocyclic lactones of microbial occurring or avermectins	Chloride channel activators	[23]
Spinosad	Macrocyclic lactones of microbial happening	Nicotinic acetylcholine receptor agonist	[20]
Fipronil	Phenylpyrazole	The antagonist of GABA- and glutamate-gated chloride channels.	[24]
Imidacloprid	Neonicotinoids	Nicotinic acetylcholine receptor agonist	[23]

*IGR* Insect growth regulator

**Fig. 1:**
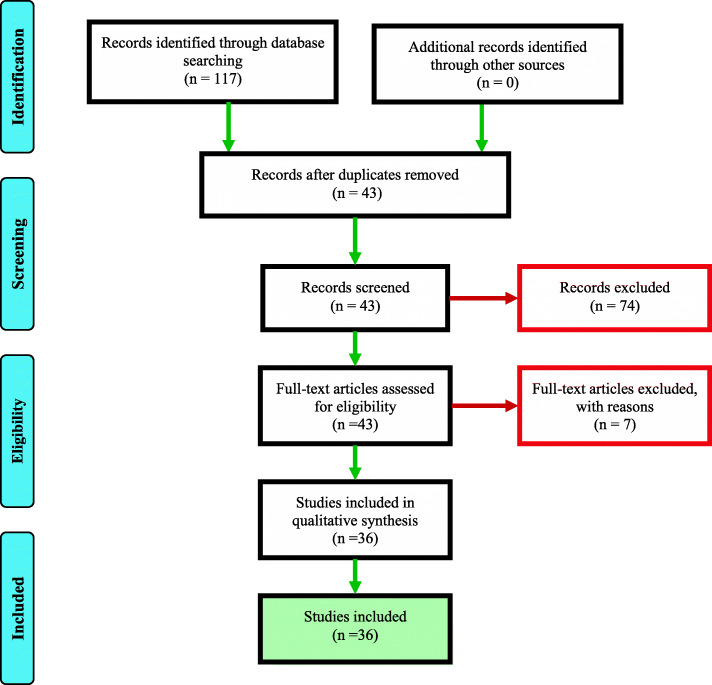
Preferred Reporting Items for Systematic Reviews and Meta-Analyses (PRISMA) flow diagram of studies included in the current review

During 1991–2019, there were 36 published papers regarding the application of systemic insecticides/endectocides for mosquito control in human and animal models (Fig. 1). In most studies (91.42%), IVM was employed against *Anopheles* species. The *Anopheles* species names and their response to endectocides and promising results are presented in Table 2 in detail. Nearly half of the studies (56.75%) were conducted on *An. gambiae* complex species. The standard dose for IVM MDA is 150 μg/kg, but different doses (from 150 μg/kg to 400 μg/kg) were assessed in several studies. Target hosts for employing systemic insecticides/drugs were animals (44.2%, including rabbit, cattle, pig, and livestock) and humans (32.35%); however, artificial membrane feeding, toxic sugar bait, and bioassays against third instar mosquito larvae were utilized for the remaining 23.45% (Table 2).

**Table 2:** The *Anopheles* species responding to endectocides and promising results in different regions

Species	Intervention	Study area	Dose	Host	Outcome
*An. gambiae* (African malaria mosquito)	IVM+AL	Burkina Faso	200 mg/kg	Human	Diminish the probability of malaria transmission by reducing the longevity
	IVM oral	London	200 mg/kg	Human	Mosquito mortality was 73%, 84%, and 89% on days 2, 3, and 4
	IVM MDA	Senegalese	150 μg/kg	Human	Reduced survival
	Eprinomectin-IVM	Lab. US	10 mg/ml	In vitro	Marginally affected re-blood feeding ability significantly increased knockdown and inhibit recovery
	IVM-AL	Burkina Faso	200 mg/kg	Human	27–35% malaria transmission reduction
	IVM	Tanzania	150–200 μg/kg	Human	47% of blood-fed died after 2 days
	IVM slow release	Tustin, California	One, two, or three silicones	Rabbit	Killing 50% up to 24 weeks
	IVM, Eprinomectin, Fipronil	Kenya	∼ 0.5 mg/kg	Cattle	Notably decreased survival
	IVM MDA	Senegal	200 mg/kg	Human	79% reduction in the mean proportion of *P. falciparum* sporozoite-infectious *An. gambiae*
*An. arabiensis*	IVM	Tanzania	0.2 mg/kg	Cattle	Decrease egg production and survival rate
	IVM and 10% sucrose	Tanzania	0.01%	Cattle	95% mortality after 48 h
	IVM, Eprinomectin, Doramectin, Moxidectin	Lab.	7.9, 8.5 ppb	Cattle	IVM and eprinomectin were lethal Doramectin reduced egg production Moxidectin was less toxic
	IVM	Semi field, Tanzania	0.2 mg/kg	Cattle	64.61% egg production reduction
*An. coluzzii*	IVM, Eprinomectin	Burkina Faso	0.2 mg/kg	Cattle	100% killed kdr mutant 3ed week after application
*An. funestus*	IVM+AL	Balonghin, Burkina Faso	200 mg/kg	Human	Diminish the probability of malaria transmission by reducing the longevity
*An. stephensi* (Asian malaria mosquito)	Avermectin	Rome	28	Mice	Highly insecticidal effect
*An. aquasalis* (Coastal areas of South America)	IVM	Amazon	200 μg/kg	In vitro/ex vivo	Declines fecundity and egg hatch rate
	IVM+CQ and IVM+PQ+CQ	Brazil and Peru	200 μg/kg	Human	84.5 and 93.6% decrease in oocyst infection prevalence and intensity
	IVM	Brazil	200 mg/kg	Human	Minimize *P. vivax* infection rate 78.33% mortality
*An. darling*i (American malaria mosquito)	IVM	Peru	200 mg/kg	Human	Minimize *P. vivax* infection rate 97.43% mortality
	IVM+CQ and IVM+PQ+CQ	Peru	5, 10, 20 and 40 ng/ml	Human	60.3 and 97% decrease in oocyst infection prevalence and intensity
	IVM	Lab	4, 8, 10, 12, 15, 20, 25*,* 30*,* 35*,* 40, 45, 50*,* 60, 65, 70 ng/ml	In vitro	Partial insecticidal effect
*An. dirus* (Asian forested zones)	IVM	Thailand	LC_25_	In vitro	44.7% oocyst prevalence reduction
*An. minimus* (South and Southeast Asia)	IVM	Thailand	LC_25_	In vitro	58.8% oocyst prevalence reduction

### *Anopheles gambiae* complex species

Application of a single oral dose of IVM (200 mg/kg) to *An. gambiae* fed on human volunteers displayed that IVM is a safe drug and can kill 89% of *An. gambiae* mosquitoes 4 days after the drug administration [25]. IVM MDA on *An. gambiae* in Southeastern Senegal considerably diminished the survivorship of mosquitoes for 6 days past the date of the MDA [26]. Among eprinomectin, selamectin, moxidectin, N-tert-butyl nodulisporamide, and IVM, only eprinomectin killed *An. gambiae* mosquitoes at concentrations close to IVM [27]. Combining IVM with antimalarial drugs (artemether-lumefantrine [AL]) in a double-blind, placebo-controlled trial lowered the likelihood of malaria transmission by *An. gambiae* after a single- or repeated-dose (200 mg/kg) treatment of IVM to 27% and 35% during the first week after treatment [28]. The effect of aging and prior blood feeding of *An. gambiae* on IVM susceptibility at 2, 6, and 14 days post-emergence (DPE) demonstrated increased susceptibility of *An. gambiae* mosquitoes (6 DPE) to IVM, particularly if they had previously been fed with blood [29]. Derua et al. (2015) assessed and compared the influence of human IVM treatment on blood-feeding *An. gambiae*. More than 47% of the blood-fed *An. gambiae* in the IVM group died after 2 days of blood feeding compared to the placebo group (97.2%).

The indirect impact of IVM MDA on malaria transmission by *An. gambiae* was studied in five villages in the Sudano-Guinean phytogeographic zone of Senegal [30]. Comparison of the rate of sporozoite in *An. gambiae* species collected from treated and untreated villages indicated a 79% reduction in the mean proportion of *P. falciparum* infection, while *P. falciparum* infection increased 246% in control villages [30]. Applying two IVM concentrations (LC_25_ and LC_5_) along with cultured *P. falciparum* NF54 at 0, 3, 6, and 9 days post parasite injection signified that the IVM administration at sub-lethal dose inhibits the sporogony of *P. falciparum* in *An. gambiae* in laboratory conditions [30, 31].

Administration of AL plus placebo or AL plus 200 μg/kg of IVM to asymptomatic *P. falciparum* carriers, *An. gambiae* and *An. funestus*, using the membrane feeding method demonstrated that IVM in combination with AL can diminish the probability of malaria transmission by reducing the longevity of blood-feeding mosquitoes in the first week after the initiation of treatment [28]. In a modeling study, the combination of IVM with AL resulted in the reduction and interruption of malaria transmission [12].

The concept of using slow-release IVM is relatively new and unique. Chaccour et al. (2015) designed and screened three different slow-release IVM formulations, including a silicone implant containing deoxycholate and sucrose. This formulation can release IVM for more than 12 weeks and is capable of killing 50% of *An. gambiae* feeding on a treated rabbit for up to 24 weeks. These observations denote that silicone-based subcutaneous formulation of IVM can safely be sustained in rabbits for up to 6 months [32]. Subcutaneous administration of slow-release IVM implant formulation in pig and cattle enhanced the level of insecticide in the host’s blood and sustained it stable for 6 months, thereby killing *An. gambiae* and *An. arabiensis* mosquitoes [33, 34].

In vitro feeding of laboratory-reared *An. arabiensis* with cattle parasiticides viz. IVM, eprinomectin, doramectin, and moxidectin revealed that IVM (LC_50_ of 7.9 ppb) and eprinomectin (LC_50_ of 8.5 ppb) were lethal to *An. arabiensis*. Doramectin markedly reduced egg development in this species. Moxidectin, however, was more than 100-fold weaker than other abovementioned insecticides, to reduce survivorship and egg production in *An. arabiensis*. Moxidectin had also a less toxic effect on *An. arabiensis* relative to the other three chemicals [35]. In Western Kenya, determination of IVM, eprinomectin, and fipronil on the survival of *An. arabiensis* disclosed that all the three compounds notably decreased the *An. arabiensis* survival [36, 37]. The effects of IVM-treated cattle on *An. arabiensis* population under the semi-field conditions in Southeastern Tanzania displayed a significant diminution in blood meal digestion, egg production (up to 15 days), and survival time (1–3 days) and also a fivefold increase in mortality in the first week [38]. Alternatively, the application of a 10% sucrose solution containing 0.01% IVM against *An. arabiensis* killed approximately 95% of mosquitoes 48 h post sugar feeding [39]. Endectocidal treatments of animals and/or humans could be a favorable new strategy for control of residual, outdoor malaria transmission. The impact of *An. arabiensis* feeding on rabbits treated with different doses of IVM on mosquito mortality exhibited that the recommended dose (1 ml/50 k) was more effective than 25% higher and 25% lower of the recommended dose in mosquito-killing mosquitos [40]. IVM-treated cattle significantly reduced egg production (64.61%) of a free-living population of *An. arabiensis* under semi-field conditions with various feeding times. The egg production rates were 54.64%,74.14%, 76.87%, and 81.62% at days 3, 6, 9, and 12, respectively, and then it decreased gradually until 15 days post-treatment [38].

Administration of IVM to cattle with an injectable therapeutic dose (0.2 mg/kg) increased the mortality of *An. coluzzii* carrying the *kdr* mutation up to 100% in the third week after the initial injection with a second blood meal of IVM [41]. Sub-lethal concentrations of IVM decreased by 33% and 36% of egg production between days 21 and 28 after injection into *An. coluzzii* fed on cattle treated with 0.2 mg/kg of IVM [41].

These results suggest that IVM and eprinomectin could be used to control zoophilic malaria vectors and reduce their population size.

### Anopheles stephensi

For the first time in 1985, two avermectin compounds, MK-933 and MK-936, were used for mosquito control [42]. To this end, 2.8 mg/l of avermectin was applied against the larvae of *An. stephensi*. When *An. stephensi* mosquitoes were fed on MK-933-treated mice and 5% sucrose solution in 0.14, 0.28, 2.8, and 28 mg/kg dosages, high insecticidal activity occurred in sucrose solution (28 mg/kg) [42].

### *An. aquasalis* and *An. darlingi*

Ex vivo evaluation of the IVM effect on *An. aquasalis* and *An. darlingi* minimized the infection rate of *P. vivax* in both species and maximized the mortality of mosquitoes (78.33% and 97.43%, respectively) 4 h post blood meal ingestion [43]. In vivo assessment of *An. aquasalis* susceptibility to IVM on three male and three female volunteers revealed that this drug could raise the mosquito mortality when blood feeding on a human host from 4 h to 14 days post-ingestion. IVM at mosquito sub-lethal concentrations (LC_5_) reduced fecundity and egg hatch rate, but not the number of pupae developed from larvae [44]. Based on a report in 2018, the oocyst infection prevalence and intensity were decreased by 60.3% and 97% in *An. darlingi* and 84.5% and 93.6% in *An. aquasalis*, respectively, when mosquitoes ingested blood from *P. vivax* patients that ingested IVM + CQ (chloroquine), PQ (primaquine) + CQ, and IVM + PQ + CQ [43].

### *An. dirus* and *An. minimus*

When IVM offered to *An. dirus* and *An. minimus* along with human *P. vivax*-infected blood, oocyst prevalence reduced by 44.7% and 58.8% in LC_25_ and 33.6% and 31.3% in LC_5_, respectively. IVM hinders the *P. vivax* sporogony development in *An. dirus* and *An. minimus* in the LC_25_ and LC_5_ concentrations [31]. Effect of IVM on *P. vivax* oocyst infection in *An. darling* in the laboratory was attributed at least partly to the insecticidal effect, as shown by the age shift in the mosquito population [31].

## Discussions

The present review has focused on the recent applications of systemic insecticides and drugs for malaria control; however, most of them were related to IVM. It could be postulated that the main goal of these studies, directly and indirectly, was focused on nine *Anopheles* malaria vectors in different zoogeographical regions including An. *gambiae*, *An*. *arabiensis*, *An. coluzzii*, *An*. *stephensi*, *An*. *aquasalis*, *An*. *dirus*, *An*. *darlingi*, *An*. *funestus*, and *An*. *minimus*. They have evaluated the efficiency of endectocides on the *Anopheles* survival rate, sporontocidal effect, reproduction rate, and malaria transmission.

IVM has been applied in different formulations and combinations such as singly, in combination with other endectocides, antimalarial drugs, sucrose, and MDA. However, their results were satisfactory and acceptable, and none of them showed 100% efficacy against different malaria vectors (Table 2). Vector control methods target the single chain of malaria transmission cycle, and candidate vaccines are still unavailable. However, for successful control goals of malaria 2016–2030, novel methods are highly needed to target the malaria transmission cycle and break its chain. In view of the fact that the current methods are inadequate for reaching the WHO Global Technical Strategy for Malaria 2016–2030, goals aim at reducing malaria mortality rates by at least 90%, controlling and eliminating malaria in at least 35 countries, and preventing the resurgence of the disease in malaria-free countries.

Taken together, candidate components of a second-generation malaria vaccine are emerging, and efforts are underway to develop a regional malaria vaccine. Moreover, simultaneously with the development of molecular markers in *Anopheles* species detection and introducing new species complexes such as *An. stephensi* [45–47], assessment of these components is crucial.

## Conclusion

It was suggested that following the WHO reports on the endectocides as vector control tools [1], a roadmap provided that ivermectin will be available as a vector control tool by 2024 [48]. Therefore, due to considerable advantages such as low price, killing the mosquito by blood feeding from the host, reduction in the sporozoite rate, endectocides, and systemic drugs could be suggested to achieve this goal. Endectocide could be suggested as a complementary intervention in malaria control and elimination programs.

Different systematic reviews of the effect of systemic insecticides on malaria disease were published. All confirmed a significant reduction in infections, though effects on the *Anopheles* survival rate, sporontocidal effect, reproduction rate, and malaria transmission, separately. The results were varied from one review to another. Near to all of the studies have been done in lab or semi-field conditions. The estimated impact on overall infection reduction was obtained after doing experiments in field conditions, a logical next step for future trials.

## Limitations

The systematic review reported here combines data across studies to estimate the effects of systemic insecticides on different malaria vectors in different zoogeographical regions. The main limitation of this systematic review, as with any overview, is that the *Anopheles* populations, the systemic insecticide application method, and regimen are not the same across studies.

## Supplementary Information

**Additional file 1:.** PRISMA 2019 checklist

**Additional file 2: Table S1**. Keywords used in the database search

**Additional file 3: Table S2**. Exclusion and inclusion criteria

## Data Availability

Data supporting this article are included within the article and supplementary file.

## Abbreviations

VBD: Vector-borne diseases
IVM: Ivermectin
IRs: Indoor residual spraying
ITNs: Insecticide-treated nets
MDA: Mass drug administration
MeSH: Medical subject heading
DPE: Days post-emergence
LC: Lethal concentrations
SIT: Sterile insect techniques
MFA: Membrane feeding assay
DFA: Direct feeding assay
AL: Artemether-lumefantrine
MEDLINE: Medical literature analysis and retrieval system online
PRISMA: Preferred reporting items for systematic review and meta-analysis
