# Supplementary material for: Endectocides as a complementary intervention in the malaria control program: a systematic review
Source: Syst Rev. 2021 Jan 18;10:30. doi: 10.1186/s13643-021-01578-9 (PMC7812718; doi:10.1186/s13643-021-01578-9)
Supplement: Supplementary file 2 — Additional file 2: Table S1. Keywords used in the database search [file 13643_2021_1578_MOESM2_ESM.doc]

| **Database** | **Keyword** | **Numbers** |
| --- | --- | --- |
| PubMed/MEDLINE | ("Malaria chemical control" OR "Malaria elimination" OR "Anopheles vector control" OR "Malaria zooprophylaxis") AND ("Systemic insecticides" OR "Endectocides" OR "Ivermectin") | 51 |
| Scopus | ("Malaria chemical control" OR "Malaria elimination" OR "Anopheles vector control" OR "Malaria zooprophylaxis") AND ("Systemic insecticides" OR "Endectocides" OR "Ivermectin") | 33 |
| Web of Science | ("Malaria chemical control" OR "Malaria elimination" OR "Anopheles vector control" OR "Malaria zooprophylaxis") AND ("Systemic insecticides" OR "Endectocides" OR "Ivermectin") | 33 |

**Supp. Table 1:** keywords used in the database search
