# Supplementary material for: Endectocides as a complementary intervention in the malaria control program: a systematic review
Source: Syst Rev. 2021 Jan 18;10:30. doi: 10.1186/s13643-021-01578-9 (PMC7812718; doi:10.1186/s13643-021-01578-9)
Supplement: Supplementary file 3 — Additional file 3: Table S2. Exclusion and inclusion criteria [file 13643_2021_1578_MOESM3_ESM.doc]

| **Inclusion** | **Exclusion** |
| --- | --- |
| English Literature | None English |
| The scope of the study  Systemic Insecticides/drugs  Endectocides  *Anopheles*  Malaria  *Plasmodium*  Human  Cattle  1991-2019 | ­2020 and later  *Culex*  *Aedes* |
| Original Electronic Article | Published just as Hard copy  Book  Congress Proceeding  Abstracts  Review Articles |

**Supp. Table 2:** Exclusion and inclusion criteria
